# Usability evaluation and adaptation of the e-health Personal Patient Profile-Prostate decision aid for Spanish-speaking Latino men

**DOI:** 10.1186/s12911-015-0180-4

**Published:** 2015-07-24

**Authors:** Donna L. Berry, Barbara Halpenny, Jaclyn L. F. Bosco, John Bruyere, Martin G. Sanda

**Affiliations:** 1 Biobehavioral Nursing and Health Systems, University of Washington, Seattle, WA USA; 2 The Phyllis F. Cantor Center, Dana-Farber Cancer Institute, Boston, MA USA; 3 Joan C. Edwards School of Medicine- Marshall University, Huntington, WV USA; 4 Department of Urology, Emory University School of Medicine, Atlanta, USA

**Keywords:** Usability, Adaptation, Spanish, Localized prostate cancer, Disparities, Decision aid

## Abstract

**Background:**

The Personal Patient Profile-Prostate (P3P), a web-based decision aid, was demonstrated to reduce decisional conflict in English-speaking men with localized prostate cancer early after initial diagnosis. The purpose of this study was to explore and enhance usability and cultural appropriateness of a Spanish P3P by Latino men with a diagnosis of prostate cancer.

**Methods:**

P3P was translated to Spanish and back-translated by three native Spanish-speaking translators working independently. Spanish-speaking Latino men with a diagnosis of localized prostate cancer, who had made treatment decisions in the past 24 months, were recruited from two urban clinical care sites. Individual cognitive interviews were conducted by two bilingual research assistants as each participant used the Spanish P3P. Notes of user behavior, feedback, and answers to direct questions about comprehension, usability and perceived usefulness were analyzed and categorized.

**Results:**

Seven participants with a range of education levels identified 25 unique usability issues in navigation, content comprehension and completeness, sociocultural appropriateness, and methodology. Revisions were prioritized to refine the usability and cultural and linguistic appropriateness of the decision aid.

**Conclusions:**

Usability issues were discovered that are potential barriers to effective decision support. Successful use of decision aids requires adaptation and testing beyond translation. Our findings led to revisions further refining the usability and linguistic and cultural appropriateness of Spanish P3P.

## Background

The Latino population is the fastest-growing minority group in the U.S. [1], and almost 13% of U.S. residents speak Spanish at home, including 5% who speak English less than “very well” [2]. Language barriers can compromise opportunities for Spanish-speaking patients in the U.S. to benefit from healthcare services afforded to English-speaking patients. According to the 2009 Healthcare Disparities Report [3], Hispanic adults reported worse provider-patient communication than non-Hispanic White adults, likely creating barriers to informed and shared decision making about health issues. The *National Action Plan to Improve Health Literacy* was published in 2010 by Health and Human Services with seven goals, including development and dissemination of accessible health information and changes in the healthcare delivery system that improve health information, communication and informed decision making [4].

Patient decision aids have been developed to support informed and shared decision making among individuals facing a variety of specific health care decisions [5]. Decision aids take a variety of formats, from printed booklets or videos to interactive websites or provider-delivered interventions. An effective decision aid will enhance patients’ decision making with information, values clarification, self-efficacy and communication skills tailored to the decision they face, resulting in a high quality decision. Most decision aids in the U.S. exist only in English, while given the growing Spanish speaking Latino population, there is an increasing need for decision support adapted to Spanish speaking patients for common health care decisions, to improve health care quality and decrease health disparities.

Shared decision making for the management of localized prostate cancer (LPC) is an appropriate area to support [6]. An estimated 233,000 incident prostate cancer diagnoses were expected in the U.S. in 2014 [7]. LPC can be treated with one or more of several modalities. While there is evidence that men diagnosed with high risk LPC are likely to live longer after curative-intent treatment [8], the majority of men are still presented management options that include active surveillance. For those considering active treatment, few findings from prospective randomized trials in North American settings adequately compare the modalities on survival or adverse outcomes such as erectile, bladder, and bowel dysfunction. Health care providers recognize that quality-of-life outcomes and a patient’s emotional and psychological considerations in addition to medical condition, are relevant to the decision [6, 9], and several investigators have reported that men with a recent diagnosis of LPC do incorporate personal preferences and factors when making treatment decisions [10–13]. However, findings that Latino and Black men are less likely to undergo prostatectomy at hospitals with robotic surgery [14], have longer time from diagnosis to surgery [15] or treatment initiation [16], and receive less medical monitoring under ‘watchful waiting’ than non-Hispanic white men [17], suggest disparities in health care access as well as in patient-provider communication, health literacy, and informed decision making.

Only two patient-centered decision aids have been shown efficacious in improving decision quality about management of localized prostate cancer in North America [18, 19]. The Personal Patient Profile – Prostate (P3P) [19, 20] is a web-based decision aid that prepares men with LPC to understand and evaluate care options, communicate personal priorities to health care providers, and make a choice consistent with personal values and medical factors. In a randomized, multisite trial in 494 U.S. English-speaking men, we found that those who used P3P experienced less decisional conflict over 6 months post-diagnosis. We undertook adapting P3P for use by Spanish-speaking Latino men through translation and usability testing to achieve a usable and linguistically and culturally appropriate Spanish decision aid.

## Methods

### Approach

Simply translating the P3P decision aid into Spanish would not be sufficient to provide usable and acceptable decision support to Spanish-speaking Latino men. Adapting P3P required in-depth evaluation of the intervention content, presentation, and technical usability by Spanish-speaking users. Our approach included preparing a candidate translation of the decision aid, and testing it with proxy users to assess usability, acceptability and needs for cultural adaptation, followed by revisions. Similar approaches are increasingly common in adapting health interventions for new target cultural and linguistic populations [21–23]. Some researchers and health communication specialists have adopted the term “transcreation” to refer to a process that incorporates translation and the creation of an new intervention adapted to the target audience [22, 24, 25].

### Translation process

Text content of the P3P was translated into Spanish using a process of forward and backward translation by three experienced, bilingual translators (one of Puerto Rican and two of Mexican nationality) working independently. Translators were asked to use terminology that would be applicable across nationalities of Spanish-speaking Latinos in the U.S. A bilingual study staff member reviewed all translations and back-translations and consulted with these translators and the principal investigator regarding particular word choice, ensuring consistency of terms in translation throughout the program. Spanish video clips were produced using bilingual Latino actors to play patients and physicians.

### Web-based decision aid

**Fig. 1:**
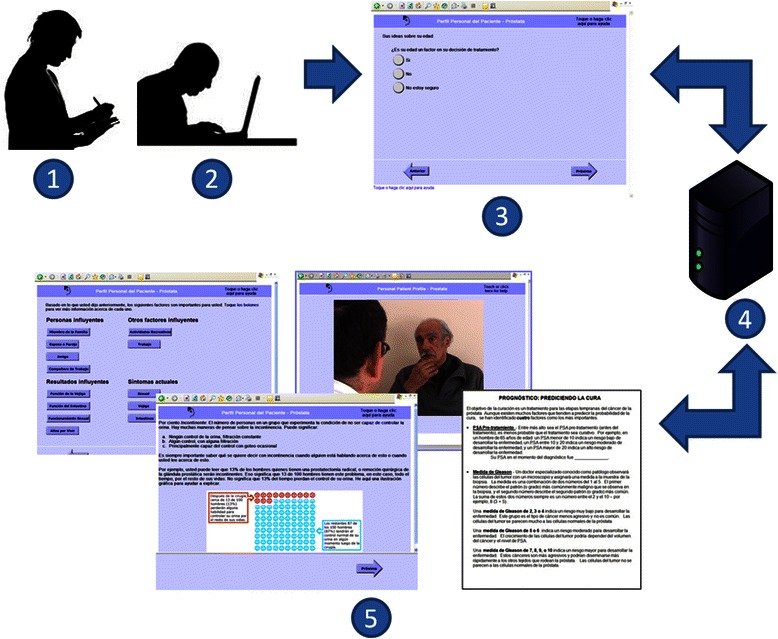
Study process and overview of the Personal Patient Profile – Prostate (P3P) web program. (1) A research assistant monitored the sessions, prompted think-aloud feedback, probed responses, and took notes. (2) Participants accessed the program on a touch screen laptop computer. (3) Questions on demographics and personal preferences were served from (4) a platform using a technical stack of Linux, Apache, MySQL, and PHP (LAMP), and participant answers were stored for re-use. (5) Answers were used to build a menu from which participants could access education on topics like understanding statistics, possible outcomes of different treatment modalities, and how to discuss priority issues with their doctors. Educational material was presented via graphs, videos, on-screen text, and nine printable teaching sheets that could be used off-line

The P3P program consists of a query component (building a “personal profile”), in which participants answer up to 32 questions about their background and personal preferences that may influence decision making for prostate cancer management, followed by an educational intervention, customized to the responses, using text, graphics and video [20] (Table 1). The web-based software initially was developed in 2004 using open–source components in a technical stack of Linux, Apache, MySQL, and PHP (LAMP) [20]. Users could view a total of 116 screens, depending on how many influential factors were reported and whether the user clicked to view additional information on each intervention topic (Fig. 1).

**Table 1:** Description of the P3P decision aid structure and content^+^

Program component	Presentation method	Purpose
*Query*		
Program introduction and instructions	• Video with a proxy patient voiceover discusses receiving a diagnosis and beginning to consider options	• Introduces user to the purpose of P3P and how to use it
	• Text instructions of how to navigate and answer questions in the website	
Questionnaires	• Page with short text introduces the subject of each questionnaire	• Tailors educational content and communication coaching to user’s values and topics of interest
	• Questionnaires cover demographics, influential decision factors, decision control preference, current symptoms, and information sources	
	• Most questions presented one question per page	
*Intervention*		
Understanding statistics	• Pictographs with text legends illustrate the percentage of patients likely to experience an outcome	• Teaches numeracy skills for understanding prostate cancer information
	• Outcomes include survival and/or possible adverse outcomes of treatment	• Provides general information on treatment outcomes
Factors influencing the care decision	• Text provides basic general information on side effects of different treatment modalities	• Teaches user treatments may have different side effects
	• Text encourages the user to talk to his doctor about concerns he endorsed – with suggested wording, including fill-in-the-blank text for the user to customize	• Coaches user to communicate values and concerns to providers to enhance education and informed decisions
	• Videos model patient-provider conversation about the selected topic	
Decision control preference	• Videos show patients with different levels of decision control discussing their care decision with their doctor	• Encourages user to think about his role in the care decision
General information about prostate cancer	• Printable teaching sheets on common topics, such as stage, care options, and side effects of treatment	• Provides general prostate cancer information the user can keep
	• Links to reputable websites	

^+^Further details and illustrations can be found in Berry et al., [20]

The user interface for the query component consisted mainly of one question per screen, with some multi-question screens, on a light-colored background. Each screen included the program logo and title, a help link and forward/backward navigation arrows. Response options were presented with large radio buttons or checkboxes. Three open-ended questions presented text boxes for responses, which required trackpad and keyboard use to navigate on the notebook computers we used in the study. The intervention component contained the same color scheme, branding, and navigation elements. Most intervention screens included text, and about one third also presented annotated infographs illustrating exemplar probabilities of various treatment outcomes, or an icon for playing a video clip that modeled a patient communicating personal concerns to a physician, with the label *Ver video (Watch video*).

### Usability testing setting and participants

Eligible participants were recruited from a genitourinary oncology clinic and a urology clinic of academic medical centers in Boston between April 2010 and July 2011. Spanish-speaking Latino men ≥ 18 years of age who had made a treatment decision for LPC within the past 2 years were eligible. Men were expected to read Spanish at a minimum 6^th^ grade reading level, assessed by self-report. Diagnoses and prostate cancer management decisions were confirmed via medical record review.

We planned a sample size of 12 participants based on Faulkner's [26] recommendation for 10–20 users in order to find 90 to 95% of usability problems in a website. All eligible subjects were invited by mail to participate in the study. Potential participants could opt-out by mail or voice message. If they did not respond within two weeks of the invitation letter being mailed, a bilingual research assistant (RA) followed-up by telephone. Participants provided written consent and received a $50 gift card in return for their time and effort. The study was approved by the Dana-Farber Cancer Institute Institutional Review Board.

### Usability sessions

Usability testing is the evaluation of information systems through testing by representative users, enabling evaluation of social acceptability, practicality, and usability of a technology [27]. We applied a cognitive interview approach to testing that included the *think aloud* method [28–30], in which participants verbally narrate their thought process while interacting with the website, plus scripted probing questions from the RA (Fig. 1).

Participants were given a verbal description of the purpose of the P3P decision aid and instructions on using a notebook touch-screen computer. The interface was accessed by touching the screen with a finger or using an attached stylus, or using the notebook keyboard for typing answers. We asked participants to imagine using the P3P when they were first diagnosed. Participants were asked to *think aloud* as they navigated the website, and when they fell silent for 20–30 seconds or became stuck in a particular task, were prompted (e.g., *Again, just tell us what you’re thinking,* or *Is there something you don’t understand?*). In addition, various pre-determined questions were asked throughout the session (e.g. *Now that you’ve read everything, is it clear what you are supposed to do next?*, *How do you understand the term ‘urinary function’?,* and *What did you think of the video?).* Participants’ behaviors and verbalizations on each screen were recorded by the RAs in handwritten field notes.

### Measures and data analysis

Usability issues and feedback were recorded by screen number. As a first step of analysis, these data were compiled and categorized using a standard coding scheme adapted from a previous usability study of P3P in English [31]. The coding scheme included three pre-determined categories: (a) navigation problems (e.g., difficulty advancing a page or playing a video), (b) content comprehension and completeness (e.g., health terminology not well understood, or desired information missing), (c) socio-cultural appropriateness (e.g., misfit of message or technology with the user’s socio-cultural background), and an emergent category, (d) proxy user problems, defined as user difficulty understanding content or performing tasks due to being a proxy, not a target, user of the intervention. Within each category, specific issues were examined and coded into unique usability sub-categories.

At the end of the testing session, participants completed a 12-item acceptability questionnaire with responses on a 5-point Likert scale (1 = lowest to 5 = highest acceptability) based on the Acceptability e-Scale [32]. Ease, helpfulness, enjoyment, time required, and overall satisfaction associated with using the program, along with specific aspects of the P3P, including infographics, video clips, prostate cancer Internet sites, and general information about prostate cancer were assessed.

We calculated the frequency of instances for each category of usability issue and summary statistics (median, minimum, maximum, mean and standard deviation) for acceptability items. Possible revisions to address the usability challenges were developed and prioritized for implementation in a revised version of P3P.

## Results

Twenty-nine Spanish-speaking Latino men with a diagnosis of LPC were contacted, and 7 men (24%) consented to participate in the study. Participant ages ranged from 54 to 67. Four of the participants reported having a college or higher degree, while 3 had not finished high school and, by observation, had significant literacy constraints. Participant characteristics are summarized in Table 2.

**Table 2:** Participant characteristics (N = 7)

	n (%)
Age	
<60 years	3 (43%)
60+ years	4 (57%)
Nationality	
Dominican	3 (43%)
Puerto Rican	1 (14%)
Salvadoran	1 (14%)
Ecuadoran	1 (14%)
Argentinean	1 (14%)
Education	
< High school	3 (43%)
College degree	2 (29%)
Postgraduate degree	2 (29%)
Language	
Monolingual	5 (71%)
Bilingual (Spanish/English)	2 (29%)
Time since prostate cancer management decision	
<1 month	1 (14%)
1-12 months	2 (29%)
13-24 months	4 (57%)
Prostate cancer management decision	
Prostatectomy	5 (71%)
Brachytherapy	1 (14%)
Active surveillance	1 (14%)

**Table 3:** Instances of usability issues by screen type.

		Usability categories				
	Number screens of each type	Navigation	Content comprehension and completeness	Socio- cultural appropriateness	Proxy user problems	Total number
**Query component**						
Program instructions	2	7	4	3	1	15
Questionnaire introductions	6	2	19	1	0	22
Pages with 1 question, 1 response allowed	40	9	58	8	3	78
Pages with > 1 question, > 1 response allowed, or combination responses	10	21	26	6	0	53
Pages with open-ended questions and text boxes for answers	3	12	13	1	0	26
**Intervention component**						
Topic selection menu *(return to this page after each topic viewed)*	2	1	0	0	0	1
Text-only pages	11	0	6	0	0	6
Topic pages with text and option buttons to view additional statistics or videos	34	10	9	1	0	20
Topic pages with text and statistics graphs	4	0	5	0	0	5
Pages with external website links	2	3	1	4	0	8
Pages to select topics and print educational information	2	0	4	1	0	5

Duration of the usability sessions ranged from 2 to 4 hours. Participants viewed an average of 80 out of 116 possible screens. A total of 239 instances of usability issues, including multiple instances of the same problem, were recorded: 65 (27%) in navigation, 145 (61%) in content comprehension and completeness, 25 (10%) in socio-cultural appropriateness, and 4 (2%) in proxy user problems (Table 3). We sub-categorized the usability issues into a total of 25 unique problems: 7 in navigation, 9 in content, 8 in sociocultural appropriateness, and 1 proxy user problem.

### Content comprehension and completeness

**Fig. 2:**
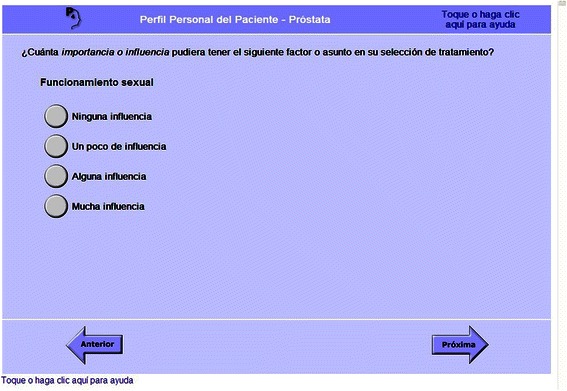
Screenshot: A set of questions on factors influencing the decision was confusing to participants. This question asked, “How much importance or influence might the following factor or issue have in your treatment choice?: Sexual function. No influence, A little influence, Some influence, A lot of influence”

The majority of usability issues were related to content comprehension. We identified 14 specific terms -- almost all from the Expanded Prostate Cancer Index Composite (EPIC-26) [33], such as *erección* (erection), *coito* (intercourse), *ha salido la orina* (urine leaking) -- that were not initially understood by participants, but were comprehended after RAs provided short definitions. We also found that a frequently used interrogatory word, *¿cuán* (how?), was not as well understood across all Spanish dialects as was an alternative, *¿que tán*. Another group of comprehension issues occurred when participants did not understand what a question was asking overall, which was most common in a set of questions about factors influencing the treatment decision (Fig. 2). These questions asked how much influence other people, lifestyle factors, or potential outcomes of treatment, such as sexual function, might have on the treatment decision. The questions were misunderstood as asking, for example, how much the cancer had influenced their sexual function, or how their current sexual function was.

In the intervention component, comprehension problems were observed among 4 participants viewing survival infographs. The participants either stated that they did not understand the pictures or offered incorrect interpretations of the figures. Other misunderstandings arose in viewing intervention text that coaches users to communicate concerns to physicians. Suggested wording was presented on screen with fill-in-the-blank spaces for the user to customize the text, for example, describing his own level of interest in maintaining sexual function. Three respondents did not understand that this text was not another question, as in the profile building component, and looked for a place to answer, or asked, *“¿Debo llenar el espacio en blanco?”* (“Do I fill in the blank?”).

### Navigation

**Fig. 3:**
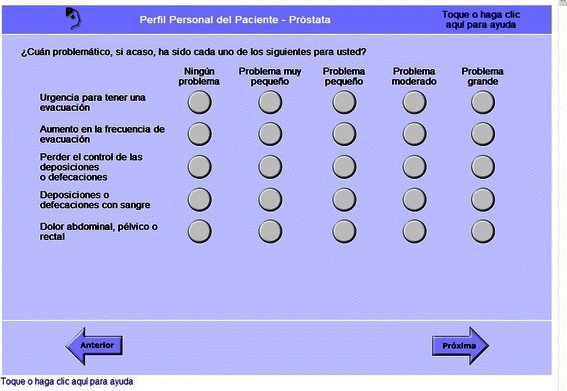
Screenshot: Multiple questions on one screen were not well understood. These questions asked, “How much of a problem, if any, is each of the following for you?: Urgency to have a bowel movement; Increased frequency of bowel movements; Losing control of your stools; Bloody stools; Abdominal/Pelvic/Rectal pain. No Problem, Very Small Problem, Small Problem, Moderate Problem, Big Problem”

**Fig. 4:**
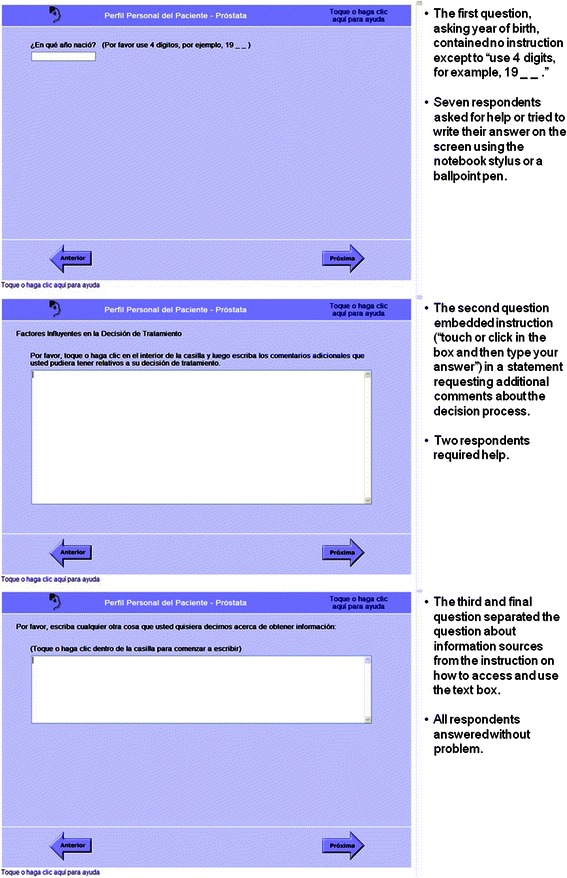
Screenshots: Three questions requiring keyboard entry of responses presented usability issues. The questions ask, “What year were you born? (Please use 4 digits, for example, 19_ _)”; “Influential factors in your treatment decision: Please touch or click inside of the box, and then type in any additional comments that you may have about your treatment decision.”; and, “Please type in anything else you would like to tell us about getting information. (Touch or click inside the box to begin typing)”

Navigation issues were the next largest category of observed usability issues. All participants experienced at least one problem answering or changing the answer to a question. This included questions that participants answered verbally, but required prompting to select an answer onscreen. The greatest difficulty observed was when multiple questions with the same response options were presented on one screen (Fig. 3); all but one respondent found the layout confusing and required help, even on subsequent screens with the same presentation. Most respondents did not know how to answer questions that required clicking inside a textbox and typing an answer using the keyboard (Fig. 4). Two participants did not understand that they could select multiple answers where response options were checkboxes rather than radio buttons, despite onscreen instruction to *Seleccione uno o más* (Select one or more). More than half of participants needed help at least one time navigating forward or backward through questions or scrolling down when not all page content was visible. Four participants did not know either how to play a video by touching or clicking an icon labeled *Ver video* (Watch video) or how to adjust the volume. Three participants did not know how to click a link to access an external website, or once on the external site, how to return to the P3P. One participant wrote down the names of linked websites rather than clicking to view them.

### Sociocultural appropriateness

A small number of comments and observations related to cultural appropriateness of the P3P and socio-technical characteristics of the study participants. Three participants stated that they either did not know how to, or did not, use the Internet. In this context, one participant characterized Latino men as being unreceptive to technology and *broncos* (stubborn); he said that Latino men would not have computers at home due to poverty and would need to use P3P in the clinics. Another participant also referred to stubbornness and *machismo* as cultural traits that perpetuate ignorance and make Latino men unwilling generally to communicate about stigmatized topics. A third participant described the topic of sexual health problems as stigmatized.

Within the demographic questionnaire, most of the men preferred only to answer the question on ethnicity and declined to answer on race, saying that no racial response option applied to them; one requested adding the response option *“mestizo”* to the race question. In the educational intervention, one respondent suggested the intervention should show statistics and data specific to Latino men with prostate cancer.

In reference to questions about role in decision making, three participants commented on family members or a priest talking to them about the decision or helping them find information, and one described faith in God as important. Four participants made comments endorsing trust in their doctors when reviewing a question about the influence of trust in doctors in decision making. As one participant stated, *‘Tienes que confiar en el médico, él es el profesional’* (‘You have to trust the doctor, he’s the professional.’). A fifth participant, however, said that *‘No creo que los médicos pueden hacer su decisión, usted debe hacer su propria decisión’* (‘I don’t think doctors can make one’s decision, you should make your own decision,’) and that the goal of the program should be to empower patients. Participants’ answers to the Control Preferences Scale [34, 35] in the P3P query, measuring their preferred level of involvement in the decision, ranged: three respondents preferred a more active role, two preferred to share the decision with their doctor, and two preferred a more passive role.

### Proxy user problems

While testing, the RAs noted and coded four instances of a usability category we refer to as proxy user problems. These cases suggested that comprehension of the study purpose and methods was complicated by the participant already having made a prostate cancer management decision and thus being a proxy user of the intervention. For example, one respondent asked whether the program was meant for those who have still not made a decision about treatment or those who already have, and suggested how the question should be re-worded for those who have already made a treatment decision. All four instances of proxy user problems occurred in the influential factors section of the profile building.

### Acceptability

Overall, the participants rated the Spanish P3P with good to high acceptability (Table 4). Three participants rated all items with the highest possible score of 5 despite having encountered observable problems navigating the program and understanding all content.

**Table 4:** Acceptability of Spanish P3P (N = 7) on 5-point scale (1 = lowest, 5 = highest)

	Median (Range)	Mean (SD)
1. How easy was the program for you to use?	4 (1–5)	3.4 (1.8)
2. How understandable were the questions?	4 (2–5)	3.7 (1.1)
3. How much did you enjoy using the program?	5 (2–5)	4.3 (1.2)
4. How helpful was it to complete the program?	5 (2–5)	4.6 (1.1)
5. Was the amount of time it took to complete the program acceptable?	5 (3–5)	4.1 (1.1)
6. How valuable was the information?	5 (2–5)	4.6 (1.1)
7. Overall, how would you rate your satisfaction with this program?	5 (2–5)	4.6 (1.1)
8. Please rate the usefulness to you of: Your part in the decision section.	5 (2–5)	4.6 (1.1)
9. Please rate the usefulness to you of: Information topics section.	5 (2–5)	4.6 (1.1)
10. Please rate the usefulness to you of: Information on statistics section.	5 (4–5)	4.9 (0.4)
11. Please rate the usefulness to you of: Video clips.	5 (1–5)	4.3 (1.6)
12. Please rate the usefulness to you of: Prostate cancer internet sites.	5 (4–5)	4.8 (0.5)

SD = standard deviation

### Revisions and re-translation

Possible revisions to address each of the 25 unique usability problems were discussed and prioritized by the research team, and most have been implemented in a revised version of P3P. Table 5 provides a sample of the problems and revisions. In addition, based on overall findings related to content comprehension, and a list of specific difficult terms, we undertook a comprehensive plain language revision of the P3P in English, with the assistance of the Dana-Farber/Harvard Cancer Center Health Communication Core. All content was re-translated by a research-focused translation team, composed of U.S.-based, native Spanish-speaking translators of Peruvian, Puerto Rican, Mexican, and Argentinean nationality, following a modified committee approach, and working from our list of key terms and the usability testing results.

**Table 5:** Selected usability issues and proposed revisions

Usability domains and program features	Problems or suggestions	Solutions or revisions
*Navigation*		
• To answer open-ended questions, users must type in a text box	• Participants did not know how to access the text box and enter answers	• Add clearer instruction
		• Put focus on the page into the text box
		• Add instruction inside the text box to *Entre su respusta aquí* (Enter your answer here)
		• Change year of birth question from textbox entry to radio buttons with a set of age categories
• Text and graphics require scrolling on some pages	• Not all participants were familiar with scrolling using the side scroll bar or down/up arrows	• Add a touch-button when needed directing the user to “go down” on the page to continue reading
*Content comprehension and comprehensiveness*		
• Presentation and order of topics	• Participants wanted definitions and further information about terms and treatment options earlier in the program	• Add pop-up glossary feature for unfamiliar terms
		• Provide expanded definition of care options the first time they are encountered
		• Revise navigation of intervention to indicate that more general information about prostate cancer will be provided after the tailored content
• Communication coaching provides text with suggested wording and fill-in-the-blanks	• Participants did not understand the coaching text, especially the fill in the blanks	• Revise the interface to visually separate the suggested wording
		• Revise the fill in the blank statements to more clearly indicate how to use them
*Sociocultural appropriateness*		
• Query about influential decision factors includes important people, lifestyle factors, current symptoms, and potential treatment outcomes	• Participant referred to God as influential in decision process	• Add a question about how religious belief or faith may influence their decision, in order to provide familiar and comprehensive decision factors
• Infographs illustrate statistics on survival and treatment side effects and teach numeracy	• One participant suggested that statistics specific to Hispanic patients be provided	• Review literature for evidence of survival/outcomes differences by ethnicity; if none found, add wording to indicate figures presented are for all ethnicities

## Discussion

Among Latino men using the Spanish P3P, usability issues were most common on screens that assessed personal factors influencing the treatment decision and current symptoms. The majority of usability issues reported were due to lack of content comprehension or translation, with a significant number of navigation problems also observed. Despite these usability issues, the participants found the Spanish P3P decision aid highly acceptable.

We identified usability issues among Spanish-speaking men similar to results of a usability study evaluating the Healthy Texas Spanish health website among medically underserved Hispanic patients [36]. Like the Healthy Texas study, we found usability issues related to unfamiliarity with Internet use, but also higher satisfaction than performance among test users. Session length was longer than we anticipated from a prior think-aloud study [31] with African American men using the English version of P3P in a community setting. In the Healthy Texas website study, lower computer literacy users required longer time than higher literacy users to engage in usability testing sessions [36].

Despite not having used the Internet previously or espousing cultural stereotypes about resistance to technology among Latinos, some participants were open to using the web-based decision aid and able to learn how to navigate the website. In the Healthy Texas usability testing [36], users initially said they were least likely to consult online resources when looking for health information, but after viewing the website, would use it again and recommend it to others. Such findings suggest that merely providing the Spanish P3P decision aid on the Internet and expecting men with prostate cancer to locate and use it on their own may be insufficient to reach all users who could benefit. Incorporating delivery of the tool at the point of care, framing it as appropriate for Latino men, and guiding them through its use may be necessary for uptake.

We found mixed evidence on a common theme in studies on Latino patient-provider communication and decision making: trust in doctors and reliance on physician decision making [21, 37]. While some participants endorsed reliance on the doctor to make a treatment decision for them, others wanted to take an active role in deciding and expressed interest in patient empowerment. Though a widely acknowledged cultural characteristic, Latino patients’ trust or respect for doctors should not be assumed to mean that even patients with limited health literacy or inability to communicate with providers in their own language lack interest in adequate information and a role in their own health care decisions. It is appropriate to prepare Spanish speaking Latino men with prostate cancer for understanding and negotiating treatment decisions for LPC.

A strength of this study was adapting a tested, effective decision aid for use by Spanish speakers and engaging end users in the iterative adaptation. Despite the large number of tested decision aids cataloged in a recent review article [5], only a small number of studies [21, 38–42] report the development and testing of decision aids for Spanish speakers in the U.S. We aimed to adapt, and not merely translate, the decision aid in a way that maintained fidelity to the intervention but was linguistically and culturally appropriate for Spanish speakers [21–23, 43]. While it may be ideal to create a Spanish intervention beginning with formative research in the priority population, it is an increasingly common approach [21, 22, 25, 42] to adapt an English language health intervention through culturally focused evaluation with a new population. In addition, working from an existing intervention in English, we were easily able to substitute Spanish text and video clips and test a fully functional interactive website, rather than only paper mockups, as may have been the case starting development of a totally new intervention.

We also note some limitations of our evaluation. First, our sample was a convenience sample with a lower response rate than anticipated, and all participants were recruited from two institutions in the Northeast. Nationalities of our participants matched those of Massachusetts and the Northeast generally, but are not representative of other Latino populations in the U.S. Second, we did not measure literacy, health literacy, nor prior computer use by participants, which reduces our ability to assess appropriate audience for the intervention.

Third, the sample included men who had already faced the decision supported by the intervention, a methodological choice both recommended [44] and commonly practiced [45–47] in assessing decision aids. However, in this study, using the decision aid and answering questions about how useful it *would have been* during the decision process was a significant challenge for some participants, likely complicating comprehension of P3P generally. It is possible that for some users, particularly those with low literacy, the intervention would be more understandable when related to a prospective, or prospective and hypothetical, treatment decision. Moreover, as Reichlin et al. [44] noted after usability testing a decision aid for LPC treatment focused on side effects, the perspective of men who have lived through the decision is likely to differ from that of the intended audience, those who have not yet made a decision.

## Conclusions

Despite some limitations, which suggest areas for further research, our usability testing and revision process represents a significant improvement over mere direct translation. The findings from our usability study suggest that the direct translation is not sufficient for successful use of decision aids in health care settings. Content comprehension of the direct translation was the most common usability problem, necessitating additional revision to plain language in Spanish, but other usability issues that related to navigation, Internet skills, and content required attention. Improvements to the Spanish P3P have been made based on the results of this evaluation in order to increase the comprehension and usability of the decision aid among Spanish-speaking Latino men with LPC.

## Abbreviation

LPC: Localized prostate cancer
